# On the insulator-to-metal transition in titanium-implanted silicon

**DOI:** 10.1038/s41598-018-22503-6

**Published:** 2018-03-07

**Authors:** Fang Liu, Mao Wang, Yonder Berencén, Slawomir Prucnal, Martin Engler, René Hübner, Ye Yuan, René Heller, Roman Böttger, Lars Rebohle, Wolfgang Skorupa, Manfred Helm, Shengqiang Zhou

**Affiliations:** 1 Helmholtz-Zentrum Dresden-Rossendorf, Institute of Ion Beam Physics and Materials Research, Bautzner Landstr. 400, 01328 Dresden, Germany; 2 Technische Universität Dresden, 01062 Dresden, Germany

## Abstract

Hyperdoped silicon with deep level impurities has attracted much research interest due to its promising optical and electrical properties. In this work, single crystalline silicon supersaturated with titanium is fabricated by ion implantation followed by both pulsed laser melting and flash lamp annealing. The decrease of sheet resistance with increasing Ti concentration is attributed to a surface morphology effect due to the formation of cellular breakdown at the surface and the percolation conduction at high Ti concentration is responsible for the metallic-like conductivity. The insulator-to-metal transition does not happen. However, the doping effect of Ti incorporation at low concentration is not excluded, which might be responsible for the sub-bandgap optical absorption reported in literature.

## Introduction

Hyperdoping Si with deep level impurities is one of the most effective approaches to form an intermediate band (IB). IB materials are candidates for infrared photodetectors and highly efficient solar cells^1–8^. The strong optical absorption by transitions in the near-infrared range from 1100 to 2500 nm has been observed in S-, Se-, and Te-hyperdoped silicon^2,9,10^. Insulator-to-metal transition (IMT) has also been reported in S- and Se-hyperdoped silicon^6,11,12^, which is a direct evidence for the formation of an IB. Titanium is another deep-level impurity candidate for forming an IB in Si^13–15^. Theoretical calculations predict that an IB can be formed in Si when Ti ions are at either interstitial or substitutional sites^6^. Indeed, Pastor *et al*. have shown that Ti impurities are located at interstitial lattice sites in Ti-hyperdoped Si fabricated by ion implantation and pulsed laser annealing^16^. On the other hand, Markevich *et al*. found evidences for the existence of substitutional Ti atoms in the Si lattice for samples implanted with Ti at very low fluences^17^. Nevertheless, Olea *et al*. observed a strong sub-bandgap absorption in Ti-implanted Si samples, suggesting the potential for optoelectronic applications^18,19^. Regarding the electrical properties of Ti-hyperdoped Si, it is believed that Ti behaves as a deep donor^20^. Several reports claim that with increasing Ti concentration a Mott-type insulator-to-metal transition occurs which is accompanied by the formation of an IB^14,15,20,21^. However, it is worth mentioning that the interface instability in form of a cellular breakdown^22,23^ occurs when the Ti doping concentration reaches 1–2 × 10^21^ cm^−3^ (2–4 at. %) during rapid solidification^24^. In a recent paper, we have also reported that the cellular breakdown takes place in Ti-implanted Si samples after pulsed laser annealing (PLA), but it can be effectively suppressed by millisecond-flash lamp annealing (FLA)^25^. The appearance of cellular breakdown prevents Ti incorporation into the single-crystalline Si matrix. Moreover, the inhomogeneous distribution of Ti impurities also complicates the interpretation of the electrical properties. Therefore, it still remains unclear whether the IMT in Ti-hyperdoped Si happens or not.

In the present work, Ti-hyperdoped Si is fabricated by ion implantation followed by PLA and FLA, respectively. We have investigated the structural and electrical properties in the fabricated Ti-hyperdoped Si samples. After FLA, the samples remain insulating even with the highest Ti implantation fluence, whereas the sheet resistance decreases with increasing Ti concentration after PLA. According to the results from conductive atomic force microscopy (C-AFM), the decrease of the sheet resistance after PLA is attributed to the percolation of Ti-rich cellular walls and not the insulator-to-metal transition due to Ti-doping.

## Results

### Recrystallization of Ti-implanted Si

**Figure 1:**
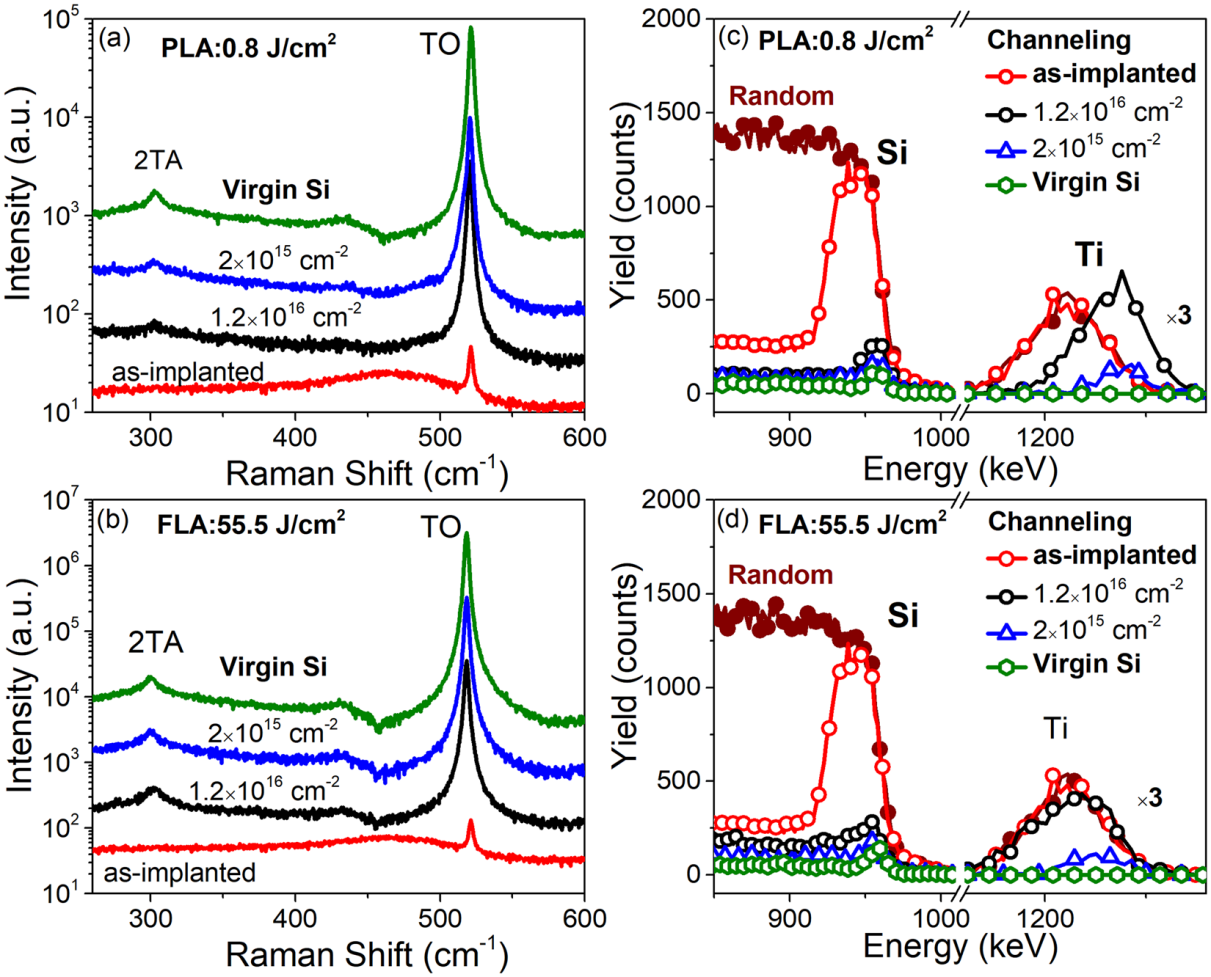
Raman scattering (**a**,**b**) and Rutherford backscattering spectrometry/channeling (cRBS) (**c**,**d**) spectra of Ti-implanted Si samples with fluences of 1.2 × 10^16^ cm^−2^ and 2 × 10^15^ cm^−2^ are shown in (**a**,**c**) after PLA at 0.8 J/cm^2^ and (b, d) FLA at 55.5 J/cm^2^. Both Raman and RBS spectra for virgin Si as well as the as-implanted samples with the fluence of 1.2 × 10^16^ cm^−2^ are shown for comparison.

Figure 1(a) and (b) show Raman spectra of Ti-implanted samples with fluences of 1.2 × 10^16^ cm^−2^ and 2 × 10^15^ cm^−2^ after PLA at an energy density of 0.8 J/cm^2^ and after FLA at 55.5 J/cm^2^, respectively. For comparison, the Raman spectra obtained from the as-implanted sample and the virgin Si are added. The Raman spectrum of virgin Si shows a narrow peak at around 520 cm^−1^ which corresponds to the transverse optical (TO) phonon mode of single-crystalline Si. A weak band at around 460 cm^−1^ is observed in the as-implanted sample due to amorphous layer formation upon ion implantation^26,27^. The weak peak at around 520 cm^−1^ for the as-implanted sample is coming from the substrate which is not affected by the ion beam. After annealing, the band associated with the amorphous layer vanishes and the strong phonon mode at 520 cm^−1^ corresponding to crystalline Si dominates the Raman spectra. The results prove that the crystal order, even in heavily implanted Si, can be well restored by both FLA and PLA.

RBS spectra obtained in both random and channeling configurations are shown in Fig. 1(c) and (d), respectively. The channeling minimum yield (χ_min_) which measures the degree of lattice damage in the host semiconductor is defined as the ratio of the backscattering yield of the channeling and the random spectra. If the implanted layer is completely damaged after implantation, χ_min_ is 100%. On the contrary, a high-quality single crystal is expected to have a χ_min_ of around 1–2%. The as-implanted sample shows a broad damage peak at around 940 keV and its χ_min_ is calculated to be about 92%, showing that the implanted region becomes amorphous. After FLA or PLA, the backscattering yields in channeling condition are very close to that of the virgin Si, pointing out that the crystal order can be restored and thus a high-lattice quality is obtained. However, the channeling backscattering yield increases slightly with increasing ion fluence from 2 × 10^15^ cm^−2^ to 1.2 × 10^16^ cm^−2^ for both PLA and FLA samples. From the RBS results, the out-diffusion of Ti after PLA is clearly observed. As shown in Fig. 1(c), the Ti peak significantly shifts towards higher energies. The detailed results have been reported in ref.^25^.

### Lattice location of Ti impurities

**Figure 2:**
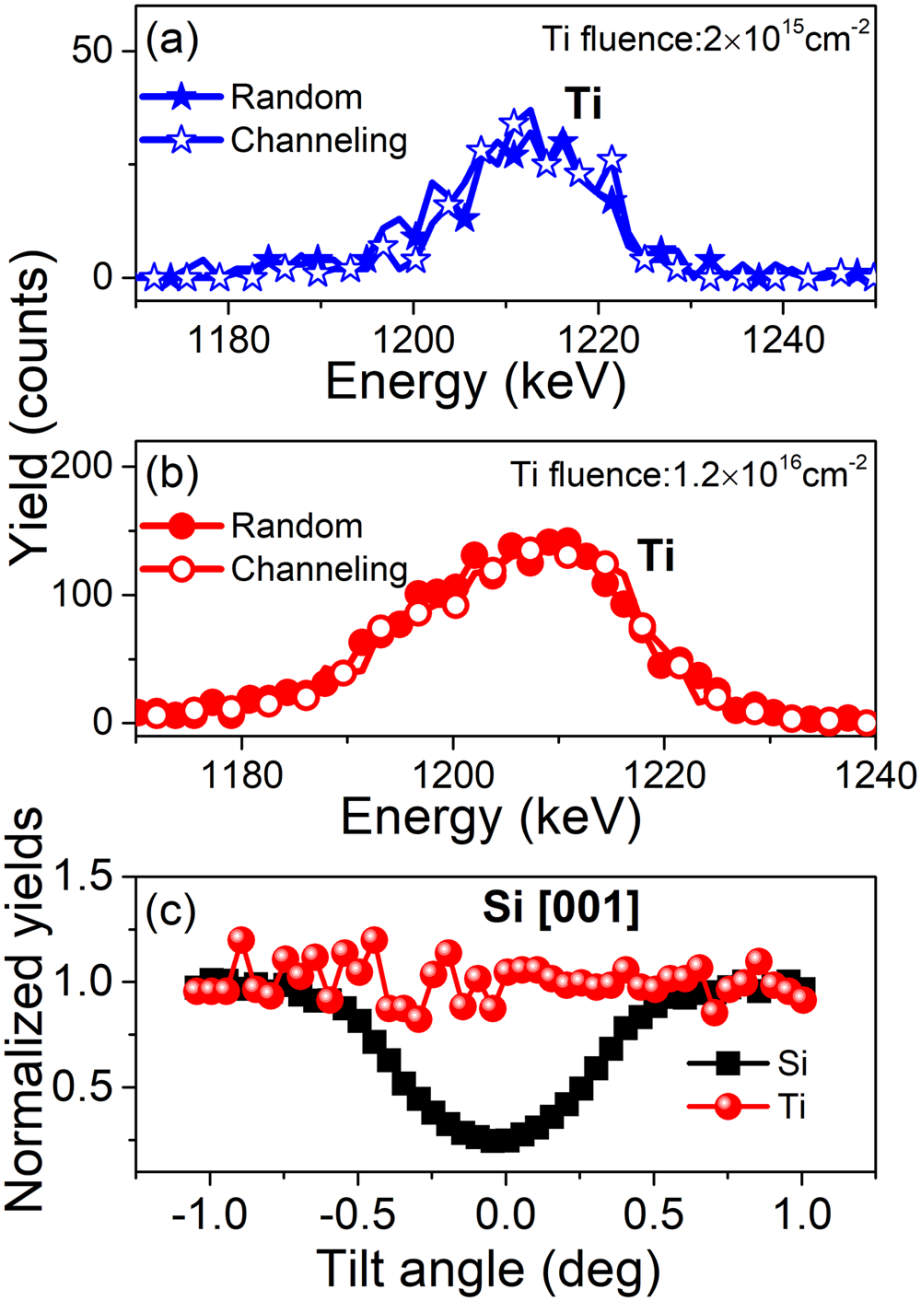
RBS spectra of Ti-implanted Si samples with fluences of 2 × 10^15^ cm^−2^ (**a**) and 1.2 × 10^16^ cm^−2^ (**b**) in random and channeling configuration for the Ti-related signal. Angular scans about the [001] direction for the Ti-implanted Si sample with a fluence of 1.2 × 10^16^ cm^−2^ (**c**). All samples shown here are flash lamp annealed.

To determine the location of the Ti atoms within the Si lattice, we show magnified RBS spectra corresponding to the Ti-related signal for the samples implanted with fluences of 2 × 10^15^ cm^−2^ Fig. 2(a) and 1.2 × 10^16^ cm^−2^ Fig. 2(b) being FLA-processed at 55 J/cm^2^. As can be seen from these figures, there are no differences between the random and channeling RBS configuration for the Ti signal, indicating that most of the Ti atoms are not located at substitutional sites. In order to further investigate the position of the Ti atoms within Si, we performed angular scans about the [001] axis of Si. The normalized scattering yield for Si and Ti atoms at the same depth is plotted in Fig. 2(c). There are significant differences between the angular scans for Ti and Si. When the sample is tilted about Si [001], the channeling behavior for Si is clearly observed, but no change is detected for Ti. This indicates that Ti atoms are not located at the substitutional sites based on the detection sensitivity of our technique^28,29^. According to cross-sectional TEM analysis, FLA leads to single-crystalline regrowth of the implanted region with the incorporation of defects, such as stacking faults. Additionally, hemispherical particles of single-crystalline nature, partially misaligned compared to the Si substrate and composed of Ti and Si are formed beneath the sample surface^25^. The RBS/channeling results can also be understood if the majority of the Ti atoms form polycrystalline secondary phases.

### Electrical properties

**Figure 3:**
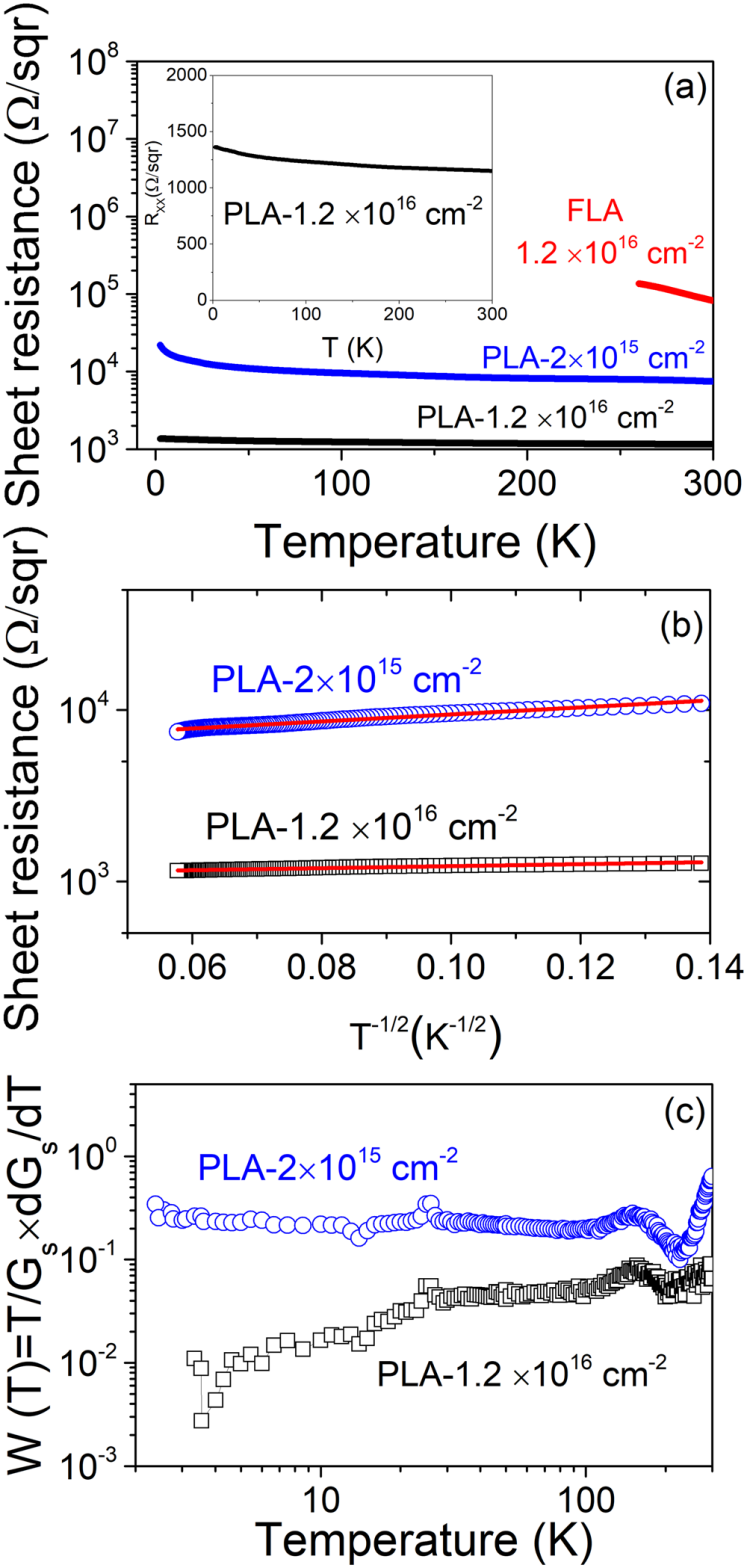
(**a**) Temperature-dependent sheet resistance of Ti-implanted Si after PLA and FLA. The inset shows the sheet resistance, in a linear scale, of the PLA-treated sample with a fluence of 1.2 × 10^16^ cm^−2^. (**b**) Analysis of transport properties for PLA samples showing the sheet resistance values as a function of *T*^−1/2^. (**c**) Reduced activation energy *W* of the conductance versus temperature for the PLA samples.

Figure 3(a) shows the temperature-dependent sheet resistance over the temperature range 2.5–300 K for samples after different annealing conditions. The inset in Fig. 3(a) shows a linear scale representation of the sheet resistance as function of temperature for the sample PLA-1.2 × 10^16^ cm^−2^. For the PLA samples, the sheet resistance decreases with increasing Ti doping. In case of samples with lower Ti concentration, the sheet resistance significantly increases with decreasing temperature, showing semiconducting behavior. However, the sheet resistance in samples with high Ti concentration shows only very weak temperature dependence [see the inset in Fig. 3(a)], similar to a metal with a low conductivity. The sheet resistance of the FLA-treated samples was found to be higher compared with the one measured in PLA-treated samples at the same implantation fluence of 1.2 × 10^16^ cm^−2^. This suggests that the electrical conductivity of those samples has a different origin. Note that the as-implanted and the virgin samples are highly resistive with a sheet resistance larger than 10^6^ Ω/sqr at room temperature.

As shown in Fig. 3(b), the temperature dependence of PLA samples can be scaled as:

1 $$R={R}_{0}exp{(\frac{{T}_{0}}{T})}^{\frac{1}{2}}$$

where ${R}_{0}$ is a pre-exponential constant and ${T}_{0}$ is a characteristic temperature related with the material properties. The characteristic parameter ${T}_{0}$ extracted from the experimental data fitting was found to be 22.6 K (∼1.96 meV) and 1.9 K (∼0.16 meV) for the sample PLA-2 × 10^15^ cm^−2^ and the sample PLA-1.2 × 10^16^ cm^−2^, respectively. This logarithmic scale of sheet resistance values as a function of *T*^−1/2^ is characteristic of granular metals when transport is governed by tunneling among isolated metal grains^30–32^, which refers to Ti cellular breakdown in the present case. Moreover, the magnitude of *T*_0_  is greatly reduced with increasing Ti implantation fluences as seen in Fig. 3(b). In comparison with the results reported in ref.^31^, the sample PLA-1.2 × 10^16^ cm^−2^ lies at the percolation threshold. In addition, Fig. 3(c) shows the reduced activation energy of the conductance at various temperatures for the PLA samples, which is derived from the potential law^33^ modelled as:

2 $$W=\frac{dln{G}_{s}}{dlnT}=(\frac{T}{{G}_{s}})\frac{d{G}_{s}}{dT}$$

where *G*_s_ is the sheet conductance deduced from the sheet resistance. As shown in Fig. 3(c), the reduced activation energy *W* is nearly zero for the sample PLA-1.2 × 10^16^ cm^−2^. This means that it exhibits a relatively finite conductivity as temperature tends to zero^34^. The decrease of *W* at low temperatures for the sample PLA-1.2 × 10^16^ cm^−2^ indicates that this sample lies in the metallic regime.

### Surface morphology

**Figure 4:**
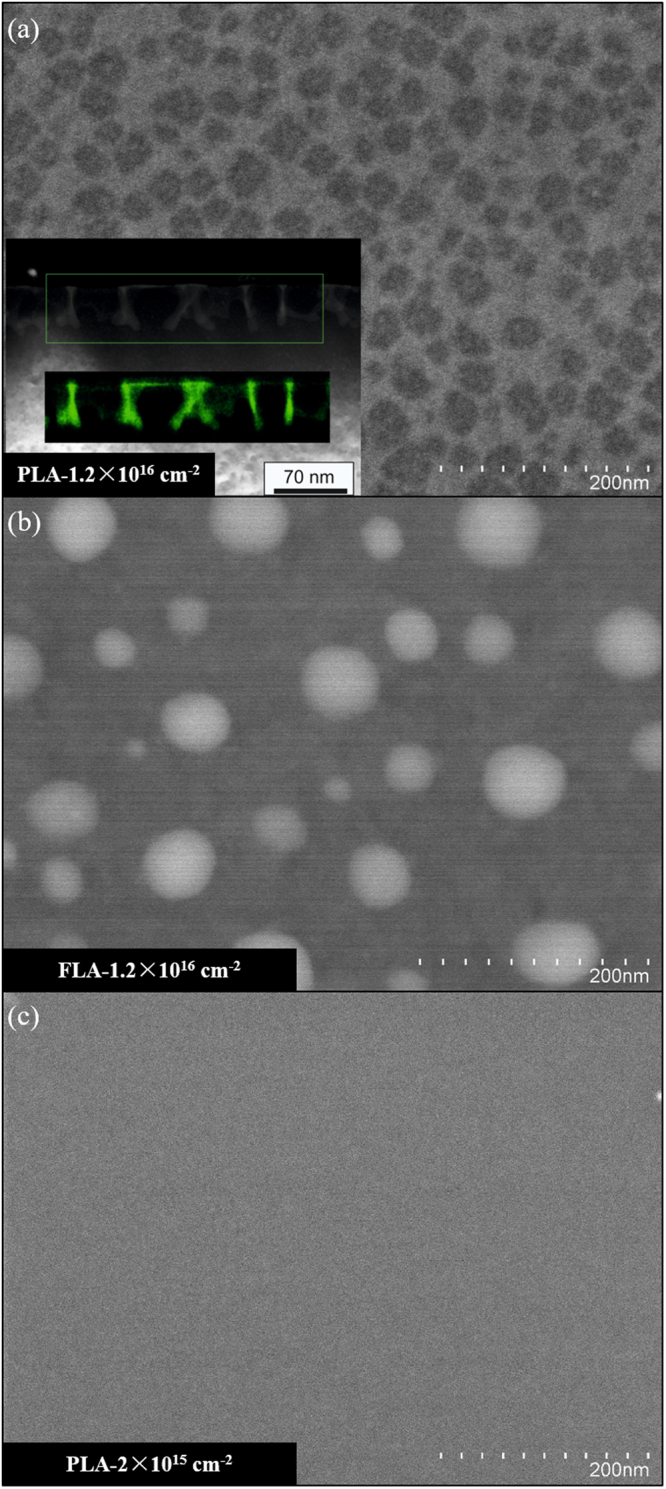
Top-view SEM image of the metallic sample PLA-1.2 × 10^16^ cm^−2^ (**a**), the insulating sample FLA-1.2 × 10^16^ cm^−2^ (**b**) and the insulating sample PLA-2 × 10^15^ cm^−2^ (**c**). The light-grey dots (sample FLA-1.2 × 10^16^ cm^−2^) and nets (sample PLA-1.2 × 10^16^ cm^−2^) are Ti-rich phases as identified by energy-dispersive X-ray spectroscopy. The inset in (**a**) displays a cross-sectional HAADF-STEM micrograph together with a Ti map (green color) obtained by energy-dispersive X-ray spectroscopy for the region marked by the green rectangle.

Figure 4(a) shows a typical SEM micrograph of Ti-implanted Si with a fluence of 1.2 × 10^16^ cm^−2^ after PLA at 0.8 J/cm^2^. Besides almost round dark-grey areas, light-grey regions are observed in the SEM micrograph. The inset in Fig. 4(a) shows the atomic-number contrast and the corresponding Ti map (green color) visualized by the high-angle annular dark-field scanning TEM (HAADF-STEM). The light-grey regions in the SEM micrograph belong to a Ti-rich cellular network structure. This network structure consists of (1) walls reaching from the sample surface to around 50 nm in depth and (2) a thin surface layer covering some of the cells formed by the walls mentioned above. According to high-resolution TEM analysis, the Ti-rich walls are characterized by an amorphous microstructure^25^. Hence, for the sample implanted at the highest fluence of 1.2 × 10^16^ cm^−2^, a complete cellular breakdown occurs at the sample surface, which is due to the occurrence of constitutional supercooling in the molten stage^23,24,35^. At high enough impurity concentrations, a morphological instability occurs at the liquid-solid interface, resulting in the lateral segregation of Ti impurities and leading to a cellular solidification microstructure. In contrast, there is no cellular breakdown occurring for FLA-treated samples even at the highest Ti fluence as shown in Fig. 4(b). The light-grey dots correspond to a Ti-rich phase and are isolated from each other. For the sample PLA-2 × 10^15^ cm^−2^, there is no detectable phase separation within the measurement sensitivity. Note that both FLA-1.2 × 10^16^ cm^−2^ and PLA-2 × 10^15^ cm^−2^ are insulating as shown in Fig. 3(a).

### Spatially resolved conduction

**Figure 5:**
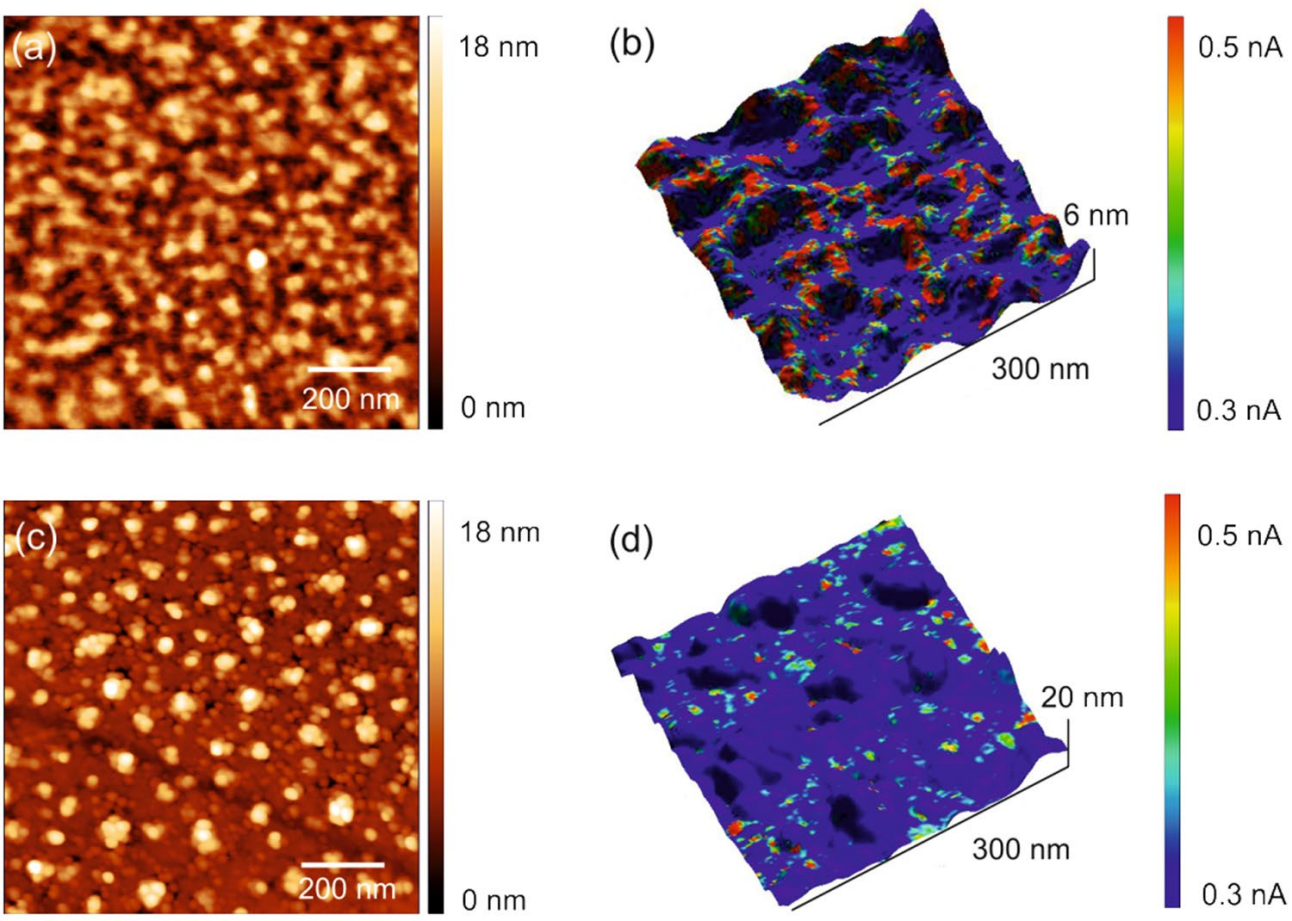
C-AFM analysis: (**a**,**c**) topography for the PLA/FLA samples, (**b**,**d**) overlay of current map and topography for the PLA/FLA samples. Both PLA and FLA sample are implanted with Ti at a fluence of 1.2 × 10^16^ cm^−2^.

The topographical images and the corresponding current maps of Ti-implanted Si samples with a fluence of 1.2 × 10^16^ cm^−2^ after PLA or FLA are shown in Fig. 5. The solidified cellular microstructure of the PLA sample can be observed in Fig. 5(a). The average cell size, determined to be ~65 nm, is slightly larger than the size obtained from TEM image (~50 nm)^25^. Instead of cellular microstructures, in Fig. 5(c), the topography of the sample after FLA shows bright spots corresponding to Ti-rich particles on the sample surface according to our TEM analysis^25^. Figure 5(b) and (d) show the overlay of current maps and topography, which provide the information about the local electrical properties of the surface layer. The current maps were measured with a sample bias voltage of +4 V. The significant dark blue areas imply current values of 0.3 nA or less. The current map shows cellular breakdown denoted as red spots in Fig. 5(b), pointing out that the electrical conductivity at the surface is not uniform. The red spots in the current map are almost connected with each other. The dark blue area shows an insulating background region. The current maps are well correlated with the topography for both samples. We can clearly observe that the conductive regions are mostly connected after PLA, whereas they are still isolated after FLA. We do believe that in Fig. 5(b) the conducting areas are associated with the cell structure formed during the rapid solidification process after PLA.

## Discussion

For the PLA-treated samples, Ti diffuses towards the surface and form Ti-rich cell walls. In case of the FLA-treated samples, however, the out-diffusion of Ti is less pronounced (see Fig. 1) and hemispherical particles of single-crystalline nature containing Ti and Si are formed beneath the sample surface^25^. For those Ti impurities beneath the surface of the FLA samples, we deduced by using cRBS technique that the majority of the Ti impurities are neither located at the substitutional nor at the interstitial sites with high symmetry. This is actually in agreement with the quantum calculations based on density-functional theory^6^. Neither interstitial nor substitutional Ti implantation process is energetically favorable, however, interstitial Ti is less unstable by 0.65 eV. Experimentally however, we do not exclude the possibility of having Ti atoms located at the interstitial site with a low symmetry^29^, i.e. Ti atoms are displaced from the symmetric interstitial site.

The change of the electrical transport properties shown in Fig. 3 may be due to the presence of percolation transition. A continuous conduction pathway spanning the sample’s dimensions is initially formed at the threshold where the percolation takes place in conducting random networks. Therefore, our results exclude the presence of a Mott-type insulator-to-metal transition in Ti-implanted Si. The metal-like conduction in samples after PLA is ascribed to the percolation of Ti-rich cell walls. Recently, atom probe tomography investigations on Co-implanted Si after PLA revealed that the phase separation does not lead to detectable silicides, but to a single-phase Si highly supersaturated with at least 10% atomic Co^36^. Moreover, our results do not exclude the fact that the infrared optical absorption reported in Ti implanted Si^18,19^ likely comes from the region of Si doped with Ti at very low concentrations, which is far below the cellular breakdown threshold. Our qualitative analysis of the absorption properties by Fourier transform infrared spectroscopy (not shown) demonstrates a near- and mid- infrared optical absorption for both PLA and FLA samples. The measured optical absorption is comparable to that reported for Ti-implanted Si infrared photodetectors^37,38^.

## Conclusion

In summary, we have investigated the structural and electrical properties of Si implanted with Ti at very high concentrations followed by either PLA or FLA. We have demonstrated that the implanted samples are recrystallized after both PLA and FLA. C-AFM results exhibit conducting networks associated with cell structures which have been formed during the rapid solidification process during PLA. Temperature-dependent Hall effect measurements have revealed that the sheet resistance decreases with increasing Ti doping concentration for the PLA samples, which is in good agreement with the percolation transition. Our results exclude the occurrence of a Mott-type insulator-to-metal transition in Ti-implanted Si. However, the dilution of Ti ions into the Si matrix at very low concentrations is not discarded, which might be responsible for the infrared optical absorption.

## Methods

Single-side polished, semi-insulating (100) Si wafers (resistivity > 10^5^ Ω·cm) with a thickness of 525 μm were implanted with Ti ions at an energy of 35 keV and fluences of 1.2 × 10^16^ cm^−2^, 6 × 10^15^ cm^−2^ and 2 × 10^15^ cm^−2^ at room temperature. In order to avoid channeling effects, samples were tilted by 7° with respect to the incident beam axis. The average thickness of the doped layer was about 60 nm^25^. After ion implantation, wafers were cut into pieces with a size of 5 × 5 mm^2^. Two annealing methods, viz. millisecond-range FLA^39^ for ms-range or PLA for ns-range were performed. The FLA was done in Ar ambient with an energy density in the range between 50 and 60 J/cm^2^, corresponding to a surface temperature between 1100 and 1300 °C. A homogeneous flash was provided by the installed array of Xe-lamps connected in parallel with a reflector behind and a pre-heating module. The PLA was carried out in ambient using a XeCl excimer laser beam (Coherent COMPexPRO201, λ = 308 nm wavelengths, 28 ns pulse duration). A homogenized 5 × 5 mm^2^ laser beam was produced with the help of a VarioLas optical system. During the PLA, a single laser pulse with an average energy density ranging from 0.2 to 0.8 J/cm^2^ was applied to irradiate the as-implanted layers.

Raman spectroscopy was performed to investigate the crystalline quality of implanted and annealed samples. The Raman scattering spectra were obtained using a 532 nm Nd:YAG laser with a charge-coupled device camera cooled with liquid nitrogen. The crystallinity of the Si matrix after annealing and the location of the Ti impurities within the Si lattice were analyzed by channeling Rutherford backscattering spectrometry (cRBS). cRBS measurements were performed with a collimated 1.7 MeV He^+^ beam at a 170° backscattering angle. The optimized energy densities for FLA and PLA were determined according to the structural properties after annealing, i.e. the narrowest Raman peak and the lowest channeling yield in RBS.

Electrical properties of implanted samples were examined using a commercial Lakeshore Hall System in van-der-Pauw-geometry between 2 and 300 K. The usage of a semi-insulating Si wafer makes the electrical measurements and data interpretation easier, since the parallel conductance from the thick insulating substrates can be neglected^20^. Scanning electron microscopy (SEM) using secondary electrons and backscattered electrons was applied to characterize the surface morphology and to qualitatively analyze the lateral compositional distribution, respectively. C-AFM was performed using tips with approx. 100 nm thick conductive diamond-like carbon coating to correlate the electrical conduction with the surface morphology.
